# Histone acetyltransferease p300 modulates TIM4 expression in dendritic cells

**DOI:** 10.1038/srep21336

**Published:** 2016-02-22

**Authors:** Bo Yang, Lin-Jing Li, Ling-Zhi Xu, Jiang-Qi Liu, Huan-Ping Zhang, Xiao-Rui Geng, Zhi-Gang Liu, Ping-Chang Yang

**Affiliations:** 1 The Center of Allergy & Immunology, Shenzhen University School of Medicine, Shenzhen, 518060, China; 2 Key Laboratory of Optoelectronic Devices and Systems of Ministry of Education and Guangdong Province, College of Optoelectronic Engineering, Shenzhen University, Shenzhen 518060, China; 3 Department of Pathology & Molecular Medicine, McMaster University, Hamilton, ON, Canada L8N 4A6; 4 ENT Institute, Longgang Central Hospital, Shenzhen 518116, China

## Abstract

TIM4 (T cell immunoglobulin mucin domain molecule-4) plays a critical role in the initiation of skewed T helper (Th) 2 polarization. The factors regulating TIM4 expression are unclear. This study tests a hypothesis that p300 and STAT6 (signal transducer and activator transcription-6) regulates TIM4 expression in dendritic cells (DC). In this study, a food allergy mouse model was developed with ovalbumin (a specific antigen) and cholera toxin (CT; an adjuvant). The chromatin immunoprecipitation assay was performed to evaluate the chromatin changes at TIM4 and STAT6 promoters. The TIM4 expression was evaluated by real time RT-PCR and Western blotting. The results showed that high levels of p300 and TIM4 were detected in the intestinal DCs of mice with intestinal allergy. p300 is involved in the CT-induced TIM4 expression in DCs. p300 interacts with the chromatin at the TIM4 promoter locus in DCs isolated from allergic mice. CT increases p300 expression to regulate STAT6 levels in DCs. STAT6 mediates the CT-induced TIM4 expression in DCs. In conclusion, p300 and STAT6 mediate the microbial product CT-induced TIM4 expression in DCs.

It is estimated that more than 20% population in the world are affected by allergic diseases^1^. The primary pathological feature of allergic reaction is the skewed T helper (Th) 2 polarization, including abnormally high levels of Th2 cytokines, such as interleukin (IL) -4, IL-5 and IL-13 and the skewed higher abundance of Th2 cells in the body^2^. Yet, the causative factors in the initiation of the skewed Th2 polarization are to be further understood.

The T cell immunoglobulin mucin domain molecule-4 (TIM4) is associated with the initiation of Th2 polarization^3^. Our previous work also observed that TIM4 played a critical role in the induction of Th2 responses and initiation of food allergy^4^. Besides, TIM4 is associated with the pathogenesis of other allergic diseases, including asthma^5^, dermatitis^6^ and allergic rhinitis^7^. TIM4 can be produced by dendritic cells (DC) after activation^8^. Our group revealed that microbial products, such as Staphylococcal enterotoxin B^4^ and cholera toxin (CT)^9^, induced DCs to produce TIM4. Yet, the mechanism by which microbial products induce the TIM4 production by DCs has not been defined.

The histone acetyltransferase p300 (also known as E1A binding protein p300, or EP300) is a protein. It is encoded by *EP300* in humans. P300 is associated with allergic diseases, such as Cui *et al.* reported that the p300-induced histone acetylation played a critical role in the induction of Th2 polarization in airway allergy^10^. Histone acetylation is an important procedure in the gene transcription. Whether p300 is involved in the expression of TIM4 has not been investigated. The signal transducer and activator transcription-6 (STAT6) is another important factor in the development of allergic reactions^11^. This protein plays a central role in exerting IL-4, the signature cytokine of Th2 responses, mediated biological responses.

STAT6 is a protein encoded by the *STAT6* gene in humans; it is one of the main factors in the regulation of the chief mediator, IgE, of allergy^12^. The cooperation between STAT6 and p300 in the pathogenesis of allergic diseases has been observed^13^,^14^. Therefore, based on the above information, we hypothesize that p300 and STAT6 modulate the expression of TIM4 in DCs. The results of the present study showed that the exposure to CT, one of the microbial products often used in the development of allergic disease animal models, significantly increased the expression of TIM4 in DCs, in which both p300 and STAT6 were associated with the regulation of TIM4 gene transcription.

## Results

### High levels of p300 and TIM4 in the intestinal DCs of mice with intestinal allergy

**Figure 1:**
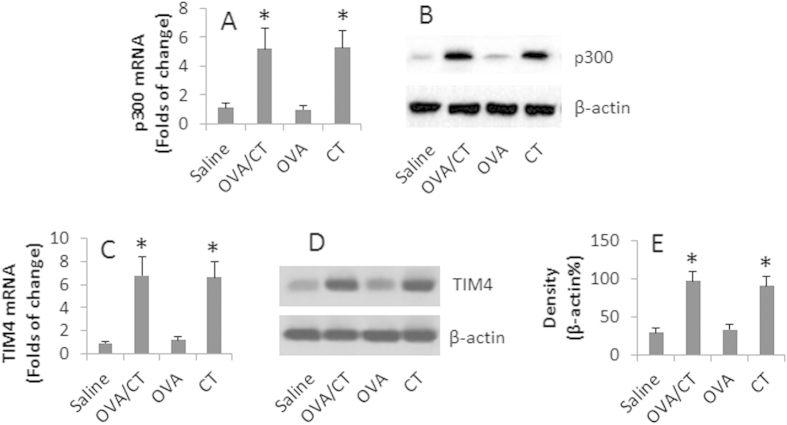
Assessment of p300 and TIM4 in DCs. BALB/c mice were treated with saline, OVA or/and CT. DCs were isolated by MACS from the small intestine of mice. The DC extracts were analyzed by RT-qPCR, Western blotting and ChIP. (**A**–**D**) The bars indicate the mRNA levels of p300 (**A**) and TIM4 (**C**) in DC extracts. The Western blots indicate the protein levels of p300 (**B**) and TIM4 (**D**) in DC extracts. (**E**) The bars indicate the summarized integrated density (measured by ImageJ) of the immune blots in panel (**D**). *p < 0.01, compared with the saline group. Each group consists of 6 mice. Samples from individual mice were analyzed separately.

Published data indicate that both p300 and TIM4 are associated with the pathogenesis of allergic reactions^3^,^15^. The relation between p300 and TIM4 in allergy has not been defined yet. In this study, we developed a food allergy mouse model. The allergic mice showed high levels of serum antigen (OVA)-specific IgE, IL-4, IL-13, profound mast cell infiltration in the intestinal mucosa, antigen-specific CD4^+^ T cell proliferation, drop in the core temperature and diarrhea (Fig. S1A–I). Since both p300 and TIM4 are associated with the pathogenesis of allergy^3^,^15^, DCs are the cells responsible for the initiation of an immune response, we assessed the levels of p300 and TIM4 in the intestinal DCs. The results showed higher levels of p300 and TIM4 in DCs isolated from the small intestine of allergic mice as compared with that from the control mice (Fig. 1A–D). The data indicate that in the allergic environment, DCs express higher levels of p300 and TIM4 in the intestine.

On the other hand, mice treated with OVA alone did not show any appreciated signs of allergic reactions in the intestine (Fig. S1). The results suggest that CT may be responsible for the change of the allergic parameters in the mice. To test this, mice were treated with CT without the presence of OVA. The results showed that the levels of total IgE, Th2 cytokines, mast cell infiltration in the intestinal mucosa were increased (p < 0.05), but significantly lower than that in mice treated with OVA/CT and challenged with OVA (Fig. S1). However, the levels of TIM4 and p300 in the intestinal DCs were almost equal to those treated with OVA/CT (Fig. 1A–D). The results suggest that CT is responsible for the increase in TIM4 and p300 in DCs.

### p300 is involved in the CT-induced TIM4 expression in DCs

**Figure 2:**
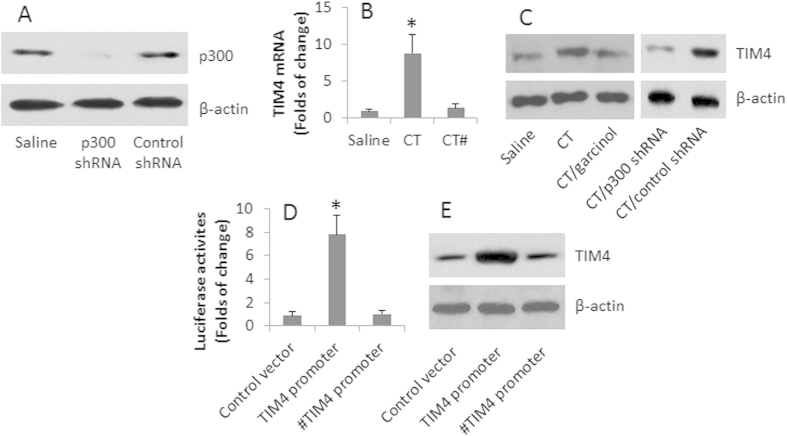
p300 is involved in the CT-induced TIM4 expression in DCs. (**A**) The immune blots show the results of p300 RNAi. (**B**–**C**) BMDCs were prepared and cultured in the presence of CT (500 ng/ml) for 48 h. The bars indicate the levels of TIM4 mRNA in DCs; the Western blots indicate the protein levels of TIM4 in DCs. Garcinol: p300 inhibitor (15 μM). (**D**–**E**) Naive BMDCs were transfected with adenovirus vectors carrying a TIM4 promoter-luciferase construct, or a control vector. The DCs were cultured in the presence of CT (500 ng/ml) for 48 h. The bars indicate the luciferase activities (**D**). The Western blots indicate the TIM4 protein levels in the DCs (**E**). The data of bars are presented as mean ± SD. *p < 0.01, compared with the saline group (**B**), or the control vector (**E**). The data are representatives of 3 independent experiments. #, in the presence of garcinol.

We next investigated the relation between p300 and TIM4 in DCs. We treated wild and p300-knockdown (Fig. 2A) BMDCs with CT in the culture for 48 h with or without the presence of a p300 inhibitor. The results showed that CT markedly induced TIM4 expression in DCs *in vitro*, while no appreciable changes of TIM4 in the presence of p300 inhibitor and CT (Fig. 2B,C). To strengthen the results, we transfected DCs with a luciferase reporter carrying the TIM4 promoter sequence (−1 to −148). The DCs were stimulated with CT in the culture for 48 h. As shown by luciferase assay, high luciferase activities were detected in the DCs (Fig. 2D). The results were further strengthened by Western blotting assay that showed higher levels of TIM4 proteins in the DCs (Fig. 2E). The results demonstrate that p300 mediates the CT-induced TIM4 expression in DCs.

### p300 binds to the chromatin at the TIM4 promoter locus in DCs isolated from allergic mice

**Figure 3:**
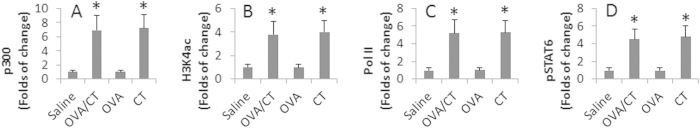
p300 interacts with the chromatin at the TIM4 promoter locus. DCs were isolated from the intestine of mice treated with the agents denoted in the figure, and analyzed by ChIP assay. The bars indicate the levels of p300 (**A**), H3K4ac (**B**), Pol II (**C**) and STAT6 (**D**) at the TIM4 promoter locus. The data of bars are presented as mean ± SD. *p < 0.01, compared with the saline group. The data are representatives of 3 independent experiments.

The data reported above implicate that p300 is involved in the TIM4 expression in DCs. We next assessed the interaction of p300 with the chromatin at the TIM4 promoter locus in DCs. DCs were isolated from the intestine of mice with or without food allergy. The DC extracts were analyzed by chromatin immunoprecipitation (ChIP). The results showed that higher levels of p300 were detected at the TIM4 promoter locus in DCs, together with the higher levels of H3K4 and RNA polymerase II (Pol II) (Fig. 3A–C). To further test the role of p300 in the CT-induced chromatin remolding at the TIM4 promoter locus, we treated wild and p300-knockdown DCs with CT in the culture for 48 h. As shown by ChIP assay, the CT-induced chromatin remolding at the TIM4 promoter locus was abolished by the deficiency of p300 (Fig. S2).

We next looked for a possible transcription factor that is responsible for the TIM4 gene transcription. We screened several Th2 response-related transcription factors, including the nuclear factor of activated T cells (NFAT), STAT6, GATA3 and E4PB4. As shown by the results of ChIP assay, high levels of STAT6 were detected at the TIM4 promoter locus in DCs of mice treated with OVA and CT or CT alone (Fig. 3D), while the NFAT, GATA3 and E4PB4 were below the detectable levels (dara are not shown).

### CT increases p300 expression to up regulate STAT6 levels in DCs

**Figure 4:**
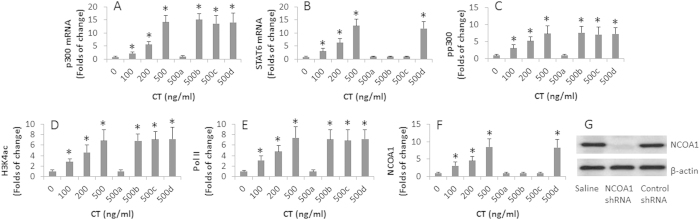
p300 mediates the CT induced-STAT6 expression in DCs. BMDCs were stimulated with CT in the culture for 48 h. The DC extracts were analyzed by RT-qPCR and ChIP assay. (**A**,**B**) The bars indicate the mRNA levels of p300 (**A**) and STAT6 (**B**) in DCs. (**A**–**D**) In the presence of garcinol (the p300 inhibitor; 15 μM; (**A**), or STAT6 inhibitor (AS1517499; 100 nM; (**B**), DCs were NCOA1-deficient (**C**) and DCs were treated with control shRNA (**D**). (**C**–**F**) DC extracts were analyzed by ChIP. The bars indicate the levels of p300 (**C**), or H3K4ac (**D**), or Pol II (**E**), or NCOA1 (**F**) at the STAT6 promoter locus. (**G**) The Western blots show the results of NCOA1 RNAi. The data of bars are presented in mean ± SD. *p < 0.01, compared with the 0 group. The data are representatives of 3 independent experiments.

The data reported above showed that after treating mice with CT, both p300 and STAT6 were increased in DCs of the mouse intestine. It seems that exposure to CT increased the expression of p300 in DCs; p300 then increased the expression of STAT6. To test this, we treated BMDCs with CT in the culture for 48 h. The results showed that CT markedly increased the mRNA levels of p300 and STAT6 in DCs in a CT dose-dependent manner (Fig. 4A,B). To test the role of p300 in the CT-increased STAT6 in DCs, in separate experiments, we added an inhibitor of p300, the garcinol, to the culture; it abolished the increase in STAT6 by CT. Treating DCs with an inhibitor of STAT6 only affected the levels of STAT6, but did not affect the levels of p300 in DCs. Meanwhile, we detected the increases in the levels of p300, H3K4, Pol II and nuclear receptor coactivator 1 (NCOA1) at the STAT6 promoter locus in DCs (Fig. 4C–F). To test if the NCOA1 acted as a transcription factor of STAT6 in DCs, we knocked down the gene of NCOA1 (Fig. 4G), and then exposed the NCOA1-deficient DCs to CT. Indeed, although the levels of p300, H3K4 and Pol II was still higher at the STAT6 promoter locus after exposure to CT in the culture, no appreciable changes of STAT6 was detected in DCs.

### STAT6 mediates the CT-induced TIM4 expression in DCs

**Figure 5:**
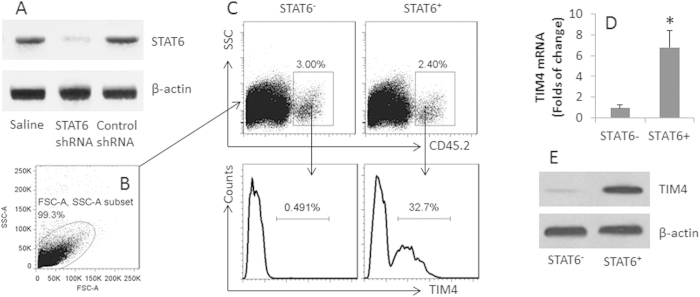
STAT6 mediates the CT-induced TIM4 expression in DCs. BMDCs were prepared from CD45.2 mice. STAT6 was knocked down in the CD45.2^+^ DCs (**A**). The STAT6^+^ and STAT6^−^ CD45^+^ DCs were adoptively transferred to CD45.1 mice; the mice were gavage-fed with CT (10 μg/mouse) daily for 4 days. LPMCs were prepared and analyzed by flow cytometry. (**B**) The gated dot plots show the cell population analyzed. (**C**) The gated dot plots show the CD45.2^+^ DCs in the LPMCs. The histograms show the frequency of TIM4^+^ CD45.2^+^ DCs. (**D**–**E**) The CD45.2^+^ DCs were isolated from LPMCs by MACS and analyzed by RT-qPCR and Western blotting. (**D**) The bars indicate the TIM4 mRNA levels (Mean ± SD; *p < 0.01, compared with the STAT6^−^ group) in the DC extracts. E, the blots indicate the TIM4 protein levels in the DC extracts. Each group consists of 6 mice. Samples from individual mice were analyzed separately. (Data from the DCs treated with control shRNA showed comparable levels of TIM4 in the naive CD45.2^+^ DCs; not shown).

We next further assessed the role of STAT6 in the CT-induced TIM4 expression in DCs. BMDCs were generated from CD45.2 mice. STAT6 was knocked down from the CD45.2^+^ DCs by RNAi (Fig. 5A) and adoptively transferred to the CD45.1 mice. The CD45.1 mice were treated with CT by gavage daily for 4 days. After sacrifice, LPMCs were isolated from the CD45.1 mice and analyzed by flow cytometry. The results showed the frequency of TIM4^+^ CD45.2^+^ STAT6^−^ DCs was below the detectable levels, while about 30% TIM4^+^ DCs were detected in those CD45.2^+^ STAT6^+^ DCs (Fig. 5B,C), or the CD45.1^+^ DCs treated with control shRNA (not shown). To strengthen the results, we isolated the CD45.2^+^ DCs from the CD45.1 mice by MACS and analyzed by RT-qPCR and Western blotting. The results showed that high levels of TIM4 were detected in the STAT6^+^ CD45. 2^+^ DCs, while only trifle levels of TIM4 were detected in the STAT6^−^ CD45. 2^+^ DCs (Fig. 5D,E).

## Discussion

TIM4 is one of the important molecules contributing to the pathogenesis of allergic diseases. By engaging with TIM1 on CD4^+^ T cells, TIM4 facilitates naive CD4^+^ T cells to differentiate into Th2 cells^3^,^4^,^9^. In the previous studies, we and others found that DCs produced TIM4 after proper stimulation^4^,^8^. Rodriguez-Manzanet *et al.* found that a subset of DCs (CD11c^+^ CD11b^+^) naturally expressed TIM4, which could be up regulated by exposure to microbial product, lipopolysaccharide^8^. Our previous studies showed that exposure to either Staphylococcal enterotoxin B or CT increased the expression of TIM4 by DCs^4^,^9^. The present study has expanded previous finding by showing mechanistic evidence that by exposure to CT, DCs expressed high levels of TIM4, in which the enrichment of p300 and STAT6 at the TIM4 promoter locus was induced by CT. The p300 and STAT6 act synergistically to promote the TIM4 gene transcription in DCs.

P300 is one of the histone acetyltransferases. This protein regulates the activity of many genes in tissues throughout the body via chromatin remodeling, and is important in the processes of cell proliferation and differentiation. It is reported that p300 activities are associated with the pathogenesis of allergic diseases. Lin *et al.* found that p300 was involved in the endothelin-1 induced VCAM-1-mediated allergic inflammation in the airway tissue^16^. Clifford *et al.* found that in the smooth muscle specimens, asthma patients had increased histone H3 acetylation, specific histone H3K18 acetylation, and increased binding of histone acetyltransferase p300 compared with nonasthmatic donors^17^. Hosogawa *et al.* observed that a role of p300 in Th2-dependent inflammation in an *in vivo* model of asthmatic inflammation, in which a Gata3/Chd4/p300 transcriptional activation complex at the Th2 cytokine loci and a Gata3/Chd4-nucleosome remodeling histone deacetylase repression complex at the Tbx21 locus in Th2 cells was defined^18^. Our data are in line with these previous studies by showing that p300 activities were significantly higher in the intestinal DCs in a food allergy animal model. Importantly, our data further demonstrated that p300 had a parallel change with TIM4 in DCs in this food allergy mouse model. The data were further supported by the *in vitro* study; exposure to CT increased both p300 and TIM4 in DCs; the increase in TIM4 was abolished by the presence of an inhibitor of p300.

A close relation was noted between p300 and STAT6, in which p300 was found to cooperate with Stat6 for induction of the STAT6-dependent transcription^13^,^14^. Gingras *et al.* suggest that the transactivation domain of Stat6 makes contact with the basal transcription machinery by binding to p300/CBP^14^. Stokes *et al.* indicate that STAT6 signaling in eosinophils is necessary for development of allergic airway inflammation^19^. In line with those pioneer studies, our data also show that STAT6 plays a critical role in the expression of TIM4 by DCs, in which Inhibition of STAT6 abolished the CT-induced TIM4 expression. Our previous studies showed that inhibition of TIM4 by a neutralizing antibody of TIM4 inhibited Th2 polarization and allergic inflammation in animal model studies^4^,^9^. Abe *et al.* also found that The therapeutic effect of anti-TIM4 mAb on arthritis^20^. The information mirrors the importance of STAT6 in the pathogenesis of Th2 polarization and the pathogenesis of allergic diseases.

The data show that, after treatment with CT alone, the serum total IgE levels and Th2 cytokines were increased in mice, indicating the Th2 polarization was induced in the mice. Whether this phenomenon is because CT facilitates other protein antigens in the intestine inducing the Th2 polarization needs to be further investigated.

In summary, the present data show that p300 and STAT6 play an important role in the CT-induced TIM4 expression in DCs.

## Materials and Methods

### Reagents

The p300 inhibitor garcinol, OVA-specific IgE ELISA kit and total IgE ELISA kit were purchased from the Biomart (Beijing, China). The ELISA kits of IL-4, IL-5, IL-13 were purchased from R&D Systems (Shanghai, China). The antibodies of p300, p300, STAT6, pSTAT6, TIM4, H3K4ac, RNA polymerase II, NCOA1, and the RNAi kits of STAT6 and NCOA1 were purchased from Santa Cruz Biotech (Shanghai, China). The immune cell isolation kits were purchased from Miltenyi Biotech (Shanghai, China). The antibodies of CD45.2 and TIM4 were purchased from BD Biosciences (Shanghai, China). The reagents for RT-qPCR and Western blotting were purchased from Invitrogen (Shanghai, China). The ChIP kit, ovalbumin and cholera toxin were purchased from Sigma Aldrich (Shanghai, China). The endotoxin levels in all reagents were detected using the Limulus assay (Limulus amebocyte lysate QCL 1000, Bio Whittaker, Walkersville, MD, USA). The reagents used in this study contained <0.2U endotoxin/10 μg reagents.

### Mice

Male BALB/c mice (6–8 week old) were purchased from the Guangzhou Experimental Animal Center. BALB/c CD45.1 and CD45.2 mice were purchased from The Jackson Laboratory (Bar Harbor, ME). Mice were housed in a pathogen–free facility with freely accessing food and water. The animal experimental procedures were approved by the Experimental Animal Ethic Committee at Shenzhen University. The experiments were carried out in accordance with the Committee guidelines.

### Development of a food allergy mouse model

BALB/c mice were gavage-fed with OAV (50 mg/kg) or/and cholera toxin (CT; 0.5 mg/kg) in 0.3 ml saline weekly for 4 weeks. The mice were orally challenged with 1 mg/mouse in week 5 and sacrificed next day. Food allergy parameters were assessed as we previously reported^9^.

### Immune cell isolation

The lamina propria mononuclear cells (LPMC) were isolated from the excised small intestine following our established procedures^21^. Immune cells were further isolated from the LPMCs with commercial reagent kits following the manufacturer’s instructions. The purity of the isolated cells was checked by flow cytometry.

### Quantitative RT-PCR (RT-qPCR)

Total RNA from T cells was extracted with Trizol (Invitrogen). The RNA (300 ng/sample) was reverse-transcribed into cDNA with a reverse transcription kit. The qPCR was performed on a real time PCR device MiniOpticon, Bio-Rad) with the SYBR Green Master Mix. The levels of mRNA were calculated by the method of 2^−ΔΔCt^. Results are presented as folds of change against the control group. The primers using in the present study include p300 (ttgtgtttcttcggcatgca and aggtggatggcaatggaaga), TIM4 (gctgcttccaacaacagtca and gtgattggatgcaggcagag) and STAT6 (gcatctatcagagggacccc and acttgtccagtcttaggccc).

### Preparation of cytosolic and nuclear extracts

Cells were incubated with lysis buffer (10 mM HEPES, pH 7.4, 10 mM NaCl, 1.5 mM MgCl2, 0.5 mM DTT, 0.2% Nonidet P-40, and 0.2 mM PMSF) at 4 °C for 15 min, and centrifuged at 500 ×g for 10 min at 4 °C. The supernatant was collected as the cytosolic extract. The pellet was added with nuclear extract buffer (20 mM HEPES-KOH, pH 7.9, 25% glycerol, 420 mM NaCl, 1.5 mM MgCl2, 0.2 mM EDTA, 0.5 mM DTT, 0.2 mM PMSF, and 1× protease inhibitor cocktail) and incubated for 15 min at 4 °C, followed by centrifugation at 13,000 ×g for 10 min at 4 °C. The supernatant was collected as the nuclear extract. The protein concentrations were determined by the Bradford method.

### Western blotting

Cell were collected and lysed in RIPA buffer (50 mM Tris-HCl, pH 8.0, 150 mM NaCl, 1% NP40, 0.5% deoxycholate, 0.1% SDS and 50 mM NaF) containing a protease inhibitor cocktail tablet. The supernatants were collected; protein concentrations were measured with a Bio-Rad DC protein assay kit. Protein lysates were diluted with loading buffer (125 mM Tris-HCl, pH 6.8, 10% β-mercaptoethanol, 4.6% SDS, 20% glycerol and 0.003% bromophenol blue) and heated at 95 °C for 5 min, fractioned by SDS-PAGE (sodium dodecyl sulfate polyacrylamide gel electrophoresis) and transferred by electroblot to a PVDF membrane. After incubation with 5% skim milk in Tris-buffered saline and 0.1% Tween 20 (TBST) for 30 min, the membranes were incubated overnight at 4 °C with primary antibodies of interest (diluted in 5% non-fat milk in TBST) and followed by incubation with peroxidase-labeled secondary antibodies for 1 h at room temperature. Washing with PBS was performed after each incubation. The immune blots on the membrane were developed with ECL (enhanced chemoluminescence). The results were photographed with an image system (UVI, Shanghai, China).

### TIM4 promoter reporter gene and luciferase Assays

A TIM4 promoter reporter gene was constructed by Promega. The construct was transfected to DCs by electroporation with a Gene Pulser II (Bio-Rad, Shanghai, China) according to the manufacturer’s instructions. The cells were then stimulated with CT in the culture for 48 h. Luciferase activity was measured and reporter activity was determined using the Dual Luciferase Reporter Assay System (Promega) according to the manufacturer’s protocol.

### Chromatin immunoprecipitation (ChIP)

ChIP was performed using a ChIP kit (Sigma Aldrich, Shanghai, China) according to the manufacturer’s instructions. The cells were fixed with fresh 1% formaldehyde for 15 min, and collected in ice-cold PBS containing protease inhibitors. The cells then were lysed in lysis buffer (50 mM Tris (pH8.1), 1% SDS, sodium pyrophosphate, β-glycerophosphate, sodium orthovanadate, sodium fluoride, EDTA, leupeptin) and the chromatin was sheared by sonicating on ice. The lysates were precleared by incubation with protein G agarose; the supernatant was harvested by centrifugation and incubated with antibodies of interest overnight at 4 °C. The immune complexes were precipitated by incubation with protein G agarose for 1 h at 4 °C. The beads were subsequently washed and eluted with elution buffer (1% SDS 0.1M NaHCO3). The DNA in the precipitated samples was recovered by reverse crosslinking at 65 °C for 4 h and analyzed by qPCR with primers of TIM4 promoter (ggaggaggtgagtcggaaat and gctgtaccactttcccttca; positions of −1 to −148) and STAT6 promoter (tctcacaaccctggaacctc and gtgctctcagaggctagagg). The data are presented as folds of change against the input (the lysates before addition of antibodies).

### Bone marrow dendritic cell preparation and culture

Bone marrow dendritic cells (BMDC) were prepared as described^22^. Briefly, BMDCs were harvested from the femurs and cultured in the BMDC medium (RPMI 1640 with 2 mM L-glutamine, 10% heat-inactivated fetal calf serum, 50 μM 2-mercaptoethanol, and 20 ng/ml granulocyte macrophage colony-stimulating factor). The medium together with reagents was changed on day 3 and day 6. Cells were collected on day 8. The CD11c^+^ DCs were isolated by MACS. The cell viability was checked by Trypan blue exclusion assay.

### RNA interference (RNAi)

NCOA1 or STAT6 gene was knocked down in DCs with commercial shRNA kits following the manufacturer’s instructions. The gene knockdown effect was checked by Western blotting 48 h after the transfection.

### Flow cytometry

Cells were stained with fluorochrome-labeled antibodies of interest for 30 min on ice. For intracellular staining, the cells were fixed and permeabilized using a Fix/Permeabilisation buffer (eBiosciences; Cat# 00-8333-56) for 1 h. After washing with PBS, the cells were incubated with fluorochrome-labeled antibodies of interest for 30 min on ice. A portion of the cells was stained with isotype IgG using as a negative staining control. The cells were analyzed with a flow cytometer (FACSCanto II). The data were analyzed with the software flowjo with the data of isotype IgG staining as a gating reference.

### *In vivo* testing the role of STAT6 in the CT-induced TIM4 expression in DCs

BMDCs were generated from the bone marrow of CD45.2 mice. The STAT6 gene was knocked down in the CD45.2^+^ DCs by RNAi. CD45.1 mice were adoptively transferred with naive CD45.2^+^ DCs, or CD45^+^ STAT6^−^ DCs, or CD45^+^ DCs treated with control shRNA, at 10^6^ cells/mouse via tail vein injection. The mice were gavage-fed with CT (10 μg/mouse in 0.3 ml saline) from next day for 4 consecutive days, and sacrificed on the fifth day. The LPMCs were isolated and analyzed by flow cytometry.

### Statistical analyses

For comparison of two groups, data were analyzed by Student’s t-test. If more than two groups, data were analyzed by one-way or two-way ANOVA with the Šidák correction for multiple comparisons. P values of <0.05 were considered statistically significant. The p value is also presented to show the levels of significance.

## Additional Information

**How to cite this article**: Yang, B. *et al.* Histone acetyltransferease p300 modulates TIM4 expression in dendritic cells. *Sci. Rep.* **6**, 21336; doi: 10.1038/srep21336 (2016).

## Supplementary Material

Supplementary Information
