# Author Correction: Histone acetyltransferease p300 modulates TIM4 expression in dendritic cells

**DOI:** 10.1038/s41598-021-89722-2

**Published:** 2021-05-11

**Authors:** Bo Yang, Lin-Jing Li, Ling-Zhi Xu, Jiang-Qi Liu, Huan-Ping Zhang, Xiao-Rui Geng, Zhi-Gang Liu, Ping-Chang Yang

**Affiliations:** 1 The Center of Allergy & Immunology, Shenzhen University School of Medicine, Shenzhen, 518060 China; 2 Key Laboratory of Optoelectronic Devices, Systems of Ministry of Education and Guangdong Province, College of Optoelectronic Engineering, Shenzhen University, Shenzhen, 518060 China; 3 Department of Pathology & Molecular Medicine, McMaster University, Hamilton, ON L8N 4A6 Canada; 4 ENT Institute, Longgang Central Hospital, Shenzhen, 518116 China

Correction to: *Scientific Reports* 10.1038/srep21336, published online 22 February 2016

This Article contains an error in Figure 5 where the data is incorrect in panel (c). The correct Figure 5 appears below as Figure 1.

**Figure 1:**
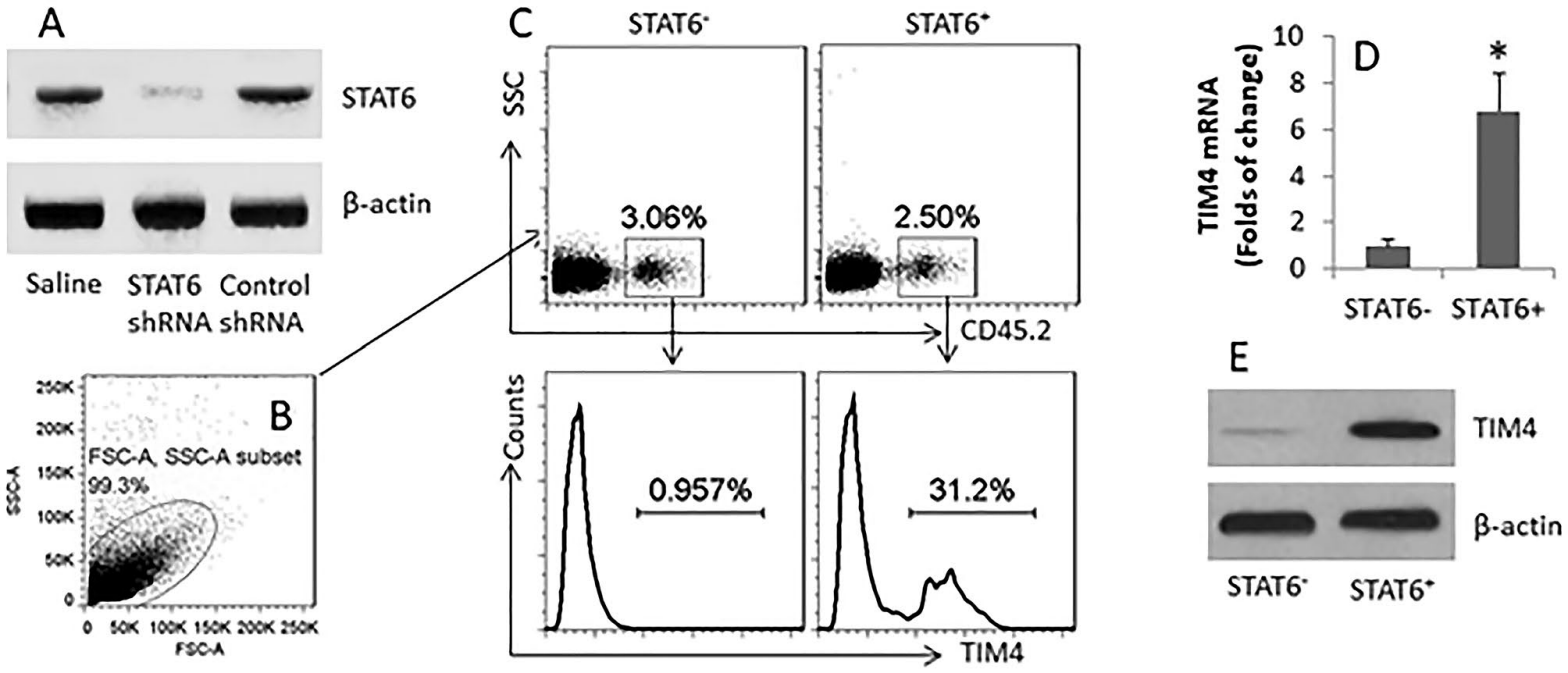
BMDCs were prepared from CD45.2 mice. STAT6 was knocked down in the CD45.2^+^ DCs (**A**). The STAT6^+^ and STAT6^−^ CD45^+^ DCs were adoptively transferred to CD45.1 mice; the mice were gavage-fed with CT (10 μg/mouse) daily for 4 days. LPMCs were prepared and analyzed by flow cytometry. (**B**) The gated dot plots show the cell population analyzed. (**C**) The gated dot plots show the CD45.2^+^ DCs in the LPMCs. The histograms show the frequency of TIM4^+^ CD45.2^+^ DCs. (**D**–**E**) The CD45.2^+^ DCs were isolated from LPMCs by MACS and analyzed by RT-qPCR and Western blotting. (**D**) The bars indicate the TIM4 mRNA levels (Mean ± SD; *p < 0.01, compared with the STAT6^−^ group) in the DC extracts. E, the blots indicate the TIM4 protein levels in the DC extracts. Each group consists of 6 mice. Samples from individual mice were analyzed separately. (Data from the DCs treated with control shRNA showed comparable levels of TIM4 in the naive CD45.2^+^ DCs; not shown).

