# Supplementary material for: Histone acetyltransferease p300 modulates TIM4 expression in dendritic cells
Source: Sci Rep. 2016 Feb 22;6:21336. doi: 10.1038/srep21336 (PMC4761958; doi:10.1038/srep21336)
Supplement: Supplementary Information [file srep21336-s1.pdf]

Supplemental materials

Histone acetyltransferase p300 modulates TIM4 expression in dendritic cells

Running title: p300 triggers TIM4 gene transcription

Bo Yang*[1,2], Lin-Jing Li*[3], Ling-Zhi Xu*[1], Jiang-Qi Liu[3,4], Huan-Ping Zhang[3], Xiao-Rui Geng[4], Zhi-Gang Liu[1], Ping-Chang Yang[1]

1, The Center of Allergy & Immunology, School of Medicine; 2, Key Laboratory of Optoelectronic Devices and Systems of Ministry of Education and Guangdong Province, College of Optoelectronic Engineering, Shenzhen University, Shenzhen 518060, China. 3, Department of Pathology & Molecular Medicine, McMaster University, Hamilton, ON, Canada L8N 4A6. 4, ENT Institute, Longgang Central Hospital, Shenzhen 518116, China.

Corresponding authors: Dr. Ping-Chang Yang and Dr. Zhi-Gang Liu. Rm722, Medical School Building, Shenzhen University, 3688 Nanhai Blvd, Shenzhen 518060, China. Email: pcy2356@szu.edu.cn and lzg@szu.edu.cn. Tel: +86-755-86671907. Fax: +86-755-86671906.

*These authors equally contributed to this work

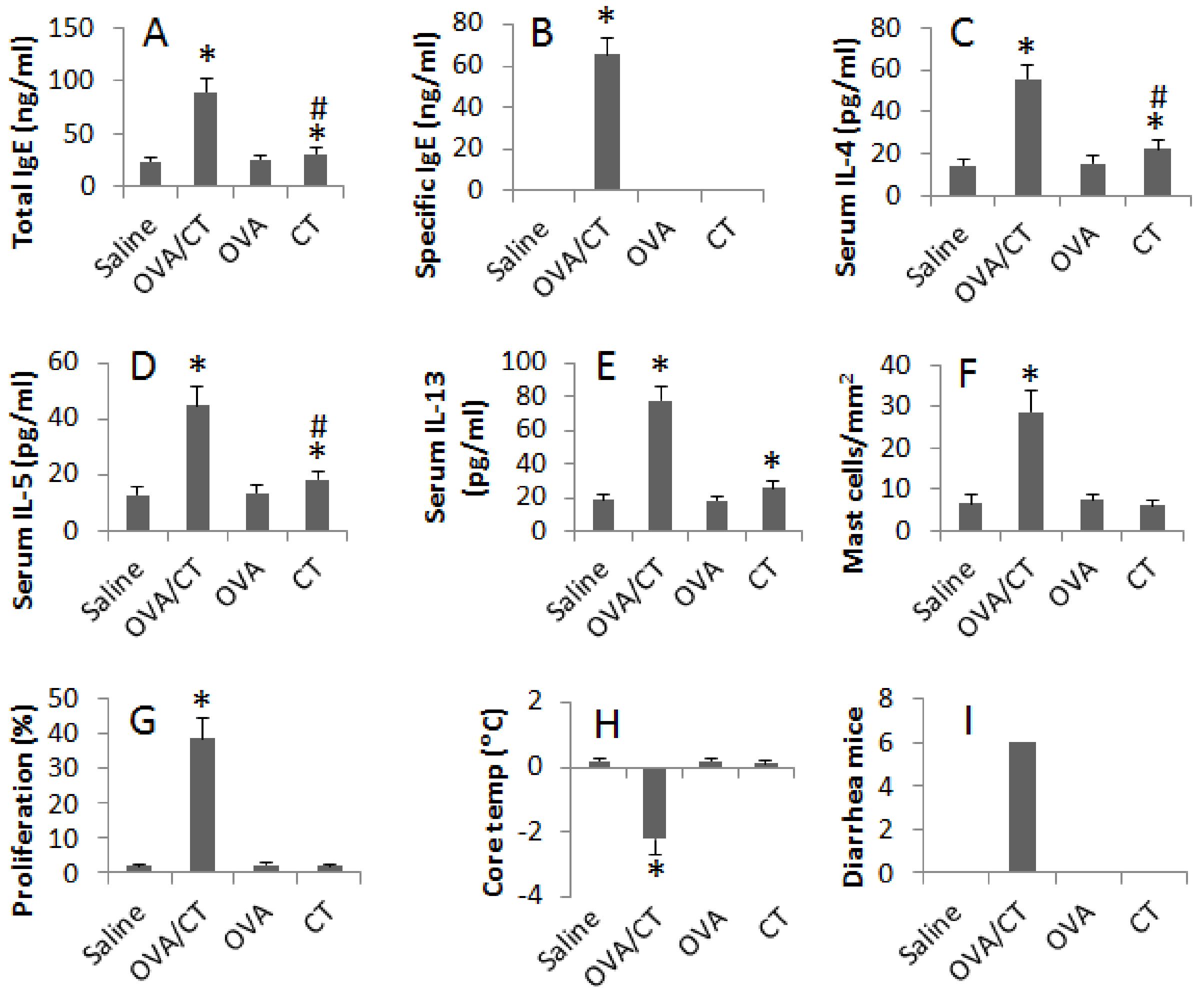

Fig. S1. Allergic reactions in the mouse intestine. BALB/c mice were treated with saline, OVA or/and CT. The bars indicate the levels of serum total IgE (A), OVA-specific IgE (B), IL-4 (C), IL-5 (D), IL-13 (E), mast cell infiltration in the intestinal mucosa (F), intestinal OVA-specific CD4+ T cell proliferation (G), core temperature drop (H) and diarrhea mice (I). Data are presented as mean ± SD. *, $p<0.01$, compared with the saline group. Each group consists of 6 mice. Samples from individual mice were analyzed separately.

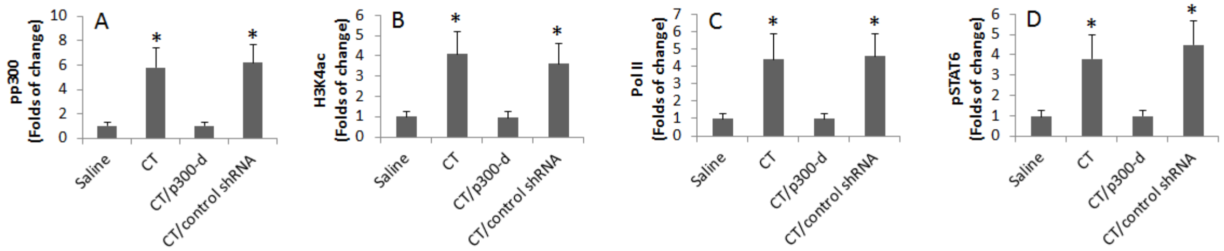

Fig. S2. P300 plays an important role in the chromatin remolding at the TIM4 promoter locus. Wild and p300-knockdown (p300-d) BMDCs were stimulated with CT in the culture for 48 h. The DCs were analyzed by ChIP. The bars indicate the levels of pp300 (A), acetylated H3K4 (H3K4ac) (B), Pol II (C) and pSTAT6 (D) at the TIM4 promoter locus in the DCs. Data are presented as mean ± SD. *, $p<0.01$, compared with the saline group. The data are representatives of 3 independent experiments.
